# Mediator of DNA Damage Checkpoint Protein 1 Facilitates V(D)J Recombination in Cells Lacking DNA Repair Factor XLF

**DOI:** 10.3390/biom10010060

**Published:** 2019-12-30

**Authors:** Carole Beck, Sergio Castañeda-Zegarra, Camilla Huse, Mengtan Xing, Valentyn Oksenych

**Affiliations:** 1 Department of Clinical and Molecular Medicine (IKOM), Norwegian University of Science and Technology (NTNU), 7491 Trondheim, Norway; 2 St. Olavs Hospital, Trondheim University Hospital, Clinic of Medicine, Postboks 3250 Sluppen, 7006 Trondheim, Norway; 3 Department of Biosciences and Nutrition (BioNuT), Karolinska Institutet, 14183 Huddinge, Sweden

**Keywords:** *V(D)J* recombination, vAbl cells, B lymphocytes, mouse genetics, genetic interaction

## Abstract

DNA double-strand breaks (DSBs) trigger the Ataxia telangiectasia mutated (ATM)-dependent DNA damage response (DDR), which consists of histone H2AX, MDC1, RNF168, 53BP1, PTIP, RIF1, Rev7, and Shieldin. Early stages of B and T lymphocyte development are dependent on recombination activating gene (RAG)-induced DSBs that form the basis for further V(D)J recombination. Non-homologous end joining (NHEJ) pathway factors recognize, process, and ligate DSBs. Based on numerous loss-of-function studies, DDR factors were thought to be dispensable for the V(D)J recombination. In particular, mice lacking Mediator of DNA Damage Checkpoint Protein 1 (MDC1) possessed nearly wild-type levels of mature B and T lymphocytes in the spleen, thymus, and bone marrow. NHEJ factor XRCC4-like factor (XLF)/Cernunnos is functionally redundant with ATM, histone H2AX, and p53-binding protein 1 (53BP1) during the lymphocyte development in mice. Here, we genetically inactivated *MDC1*, *XLF*, or both *MDC1* and *XLF* in murine vAbl pro-B cell lines and, using chromosomally integrated substrates, demonstrated that MDC1 stimulates the V(D)J recombination in cells lacking XLF. Moreover, combined inactivation of *MDC1* and *XLF* in mice resulted in synthetic lethality. Together, these findings suggest that MDC1 and XLF are functionally redundant during the mouse development, in general, and the V(D)J recombination, in particular.

## 1. Introduction

In mammalian cells, DNA double-strand breaks (DSBs) activate the DNA damage response signaling (DDR). During DDR, Ataxia telangiectasia mutated (ATM) protein kinase phosphorylates multiple substrates, including histone H2AX and the scaffold proteins, mediator of DNA damage checkpoint protein 1 (MDC1) and p53-binding protein 1 (53BP1) [1]. The E3 ubiquitin ligases, really interesting new gene (RING) finger (RNF) 8 and RNF168, function downstream of the ATM to enhance 53BP1 binding, which, in turn, facilitates the recruitment of DDR effectors, Pax transactivation domain-interacting protein (PTIP), and Rap1-interacting factor 1 (RIF1) [1]. Moreover, methylated [2,3,4] and acetylated [5] histones may facilitate the DDR. In particular, histone H4 lysine 20 di-methylation (H4K20me2) [3] and histone H3 lysine 79 mono- and di-methylation (H3K79me1/2) [4] were thought to facilitate recruitment of 53BP1 to the sites of damaged DNA. Homologous recombination (HR), classical non-homologous end joining (NHEJ), and alternative end joining (A-EJ) are cellular pathways that recognize and repair DSBs. NHEJ is initiated by the recruitment of the core Ku70/Ku80 (Ku) sensor to the DSB sites. Ku facilitates the recruitment of downstream factors, including the DNA-dependent protein kinase, catalytic subunit (DNA-PKcs), and the NHEJ core factors DNA ligase 4 (Lig4) and X-ray repair cross-complementing protein 4 (XRCC4). A number of NHEJ proteins, including accessory factors, stabilize the DNA repair complex and process DNA overhangs to facilitate ligation [1]. Among them, nuclease Artemis [6], XRCC4-like factor (XLF, or Cernunnos) [7,8], a paralogue of XRCC4 and XLF (PAXX) [9,10,11], and modulator of retrovirus infection (Mri) [12,13].

During the B and T lymphocyte development, both DDR and NHEJ pathways function in response to the recombination activating gene (RAG)-induced DSBs in the process known as the variable (V), diversity (D) and joining (J) gene segments recombination (V(D)J recombination). RAG is the nuclease that generates DSBs adjacent to the *V*, *D*, and *J* gene segments of immunoglobulin and T cell receptor genes. NHEJ is the only known process to recognize and efficiently repair RAG-induced DSBs [1,14]. V(D)J recombination is ablated in mice lacking core NHEJ factors, Ku70 [15] and Ku80 [16]. Inactivation of *XRCC4* or *Lig4* resulted in embryonic lethality in mice, while conditional inactivation or knocking down of *XRCC4* or *Lig4* in lymphocytes blocked the V(D)J recombination and NHEJ [1,17,18]. Accessory NHEJ factors DNA-dependent protein kinase, catalytic subunit (DNA-PKcs) and Artemis are required for the V(D)J recombination-associated DNA repair. Artemis is a nuclease that processes RAG-induced hairpin-sealed DNA ends, and DNA-PKcs is required to both structurally stabilize and phosphorylate Artemis [6,19,20,21,22,23]. On the contrary, germline inactivation of *XLF* [24,25], *PAXX* [26,27,28,29], or *Mri* [12,13] had no or modest impact on the DNA repair and lymphocyte development in general, and the V(D)J recombination in particular. Combined inactivation of XLF and PAXX resulted in the V(D)J recombination defect in cells [30,31,32] and synthetic lethality in mice [26,28,29,33]. Moreover, XLF is functionally redundant with DNA-PKcs [33,34,35], Mri [12,13], and RAG2 [36].

DDR factors were thought to be dispensable for the V(D)J recombination, because germline inactivation of *ATM* [37], *H2AX* [38,39], *MDC1* [40], or *53BP1* [41] resulted in modest or no effect on early stages of B and T lymphocyte development. Strikingly, combined inactivation of *XLF* and *ATM* [42], or *XLF* and *53BP1* [43,44], resulted in live-born mice with nearly no mature B and T lymphocytes due to the impaired V(D)J recombination. Additional ATM-dependent DDR factors, including MDC1, may be involved in the V(D)J recombination, and their functions might be revealed in the *XLF*-deficient background [1,42,43,44].

XLF is the NHEJ factor. Mutations in the *XLF* gene in humans result in combined immunodeficiency [8,45], and inactivation of the *XLF* gene in mice results in a modest reduction of B and T lymphocytes count [24,25]. XLF shares a structure with XRCC4, and binds XRCC4 to stimulate the Lig4 activity [7]. XLF has a yeast homolog Nej1 that also stimulates the DNA repair in yeast [46]. Moreover, the lack of XLF results in increased levels of medulloblastoma in *Trp53*-deficient mice [24]. Together, these observations place XLF to the group of “core” NHEJ factors. MDC1 is a DNA damage response protein acting downstream of ATM and upstream of 53BP1 [47]. Like XLF, the MDC1 has no enzymatic activity and likely stabilizes the DNA repair complex and facilitates the recruitment of other DNA repair factors. Both MDC1 and XLF can be phosphorylated by ATM and likely by DNA-PKcs to regulate their functions in DNA repair [1]. Moreover, both XLF and MDC1 were proposed to tether the DNA at the DSB sites before the DNA ligation [1,48].

Here, we generated *MDC1^−/−^XLF^−/−^* double-knockout cell lines and demonstrated that MDC1 is stimulating the V(D)J recombination in cells lacking XLF. Moreover, we demonstrated that combined inactivation of *MDC1* and *XLF* resulted in synthetic lethality in mice.

## 2. Materials and Methods

### 2.1. Generation of Abelson Murine Leukemia Virus-Transformed (vAbl) Cell Lines

*Eμ-Bcl2^+^* and *XLF^−/−^Eμ-Bcl2^+^* vAbl cells were published earlier [34,42,43]. Five independent clones of *MDC1^−/−^Eμ-Bcl2^+^* were generated using two three-week-old mice following the procedure described previously [34,42,43,49,50]. Additionally, the *XLF* gene was inactivated in *Eμ-Bcl2^+^* vAbl cells to obtain *XLF^−/−^Eμ-Bcl2^+^* cell lines, and in *MDC1^−/−^Eμ-Bcl2^+^* to generate *MDC1^−/−^XLF^−/−^Eμ-Bcl2^+^* vAbl lines, using the clustered regularly interspaced short palindromic repeats (CRISPR)/CRISPR-associated protein 9 (Cas9) gene-editing approach as described earlier [51]. Briefly, oligonucleotides corresponding to single guide RNAs (sgRNAs) were cloned into the plasmid vector LentiCRISPR v2 (Addgene plasmid #52961, Addgene, Watertown, MA, USA) [52]. The following sgRNAs were used to target *exon 3* of the *XLF* gene: sgRNA1_FWD: 5′-CTTAGCATACACCAACTTC-3′; sgRNA1_REV: 5′-GAAGTTGGTGTATGCTAAG-3′; sgRNA2_FWD: 5′-CCACCAACAGGTACTCATA-3′; sgRNA2_REV: 5′-TATGAGTACCTGTTGGTGG-3′. Parental vAbl cells were transduced with lentiviral vectors containing corresponding sgRNA sequences, and up to 200 clones were screened by western blot. The cells lacking the XLF signal were used to validate the deletion of the *exon 3* by DNA sequencing (available upon request). Two *XLF^−/−^* clones and four *MDC1^−/−^XLF^−/−^* clones were used for experiments. Mock-treated and parental vAbl cells were used as DNA repair-proficient controls.

### 2.2. Antibodies

The following antibodies were used for western blot: rabbit polyclonal anti-XLF (Bethyl, Montgomery, TX, USA; A300-730A, dilution 1:2000), swine polyclonal anti-rabbit immunoglobulin-horseradish peroxidase-conjugated (Ig-HRP; Dako antibodies, Dako, Glostrup, Denmark; #P0399, dilution 1:5000), mouse monoclonal anti-β-actin (Abcam, Cambridge, UK; ab8226, dilution 1:2000), rabbit polyclonal anti-mouse Ig-HRP (Dako antibodies, Dako, Glostrup, Denmark; #P0260, dilution 1:5000), and goat polyclonal anti-mouse Ig-HRP (Dako antibodies, Dako, Glostrup, Denmark; #P0447, dilution 1:5000).

### 2.3. Variable (V), Diversity (D) and Joining (J) Gene Segments Recombination (V(D)J Recombination) Assays Based on Chromosomally Integrated pMX Cassettes

V(D)J recombination assays were performed using chromosomally-integrated *pMX inversion* (*pMX-INV*) and *pMX deletion* (*pMX-DEL*) substrates, as previously described [34,42,43,49,50]. In the *pMX-INV* cassette, the *green fluorescent protein* (*GFP*) gene is placed in the reversed orientation and the GFP protein is not expressed. Upon the RAG-induced V(D)J recombination, the *GFP* gene is placed in the sense orientation leading to the GFP protein expression. The GFP protein is then detected by flow cytometry to estimate the V(D)J recombination efficiency in indicated vAbl cells [42,49,50]. For the Southern blot-based experiments, we used chromosomally-integrated *pMX-DEL* cassettes. During the V(D)J recombination, the *pMX-DEL^CJ^* cassette results in an intermediate product with hairpin-sealed coding ends that require Artemis nuclease activity to open the hairpins prior DNA ligase 4-dependent DNA ligation, leading to coding joints (CJ). On the contrary, the *pMX-DEL^SJ^* cassette results in the RAG-dependent generation of blunt signal ends (SE) that can be directly ligated by DNA ligase 4 and do not require Artemis nuclease activity, leading to signal joints (SJ) [34,42,43,49,50].

### 2.4. Mice

All experiments involving mice were performed according to the protocols approved by the Norges teknisk-naturvitenskapelige universitet (NTNU), FOTS#8319. *MDC1^+/−^* [40], *XLF^+/Δ^* [24], and *Eμ-Bcl2^+^* [53] mice were described previously. The *Eμ-Bcl2^+^* transgenic mice were used to generate vAbl pre-B cells and increase cell survival during the experimental procedures [49].

### 2.5. Proliferation Assay

Fifty thousand vAbl cells were plated in 2 mL of Roswell Park Memorial Institute (RPMI) medium in triplicates into 6-well plates. Similarly, fifty thousand human haploid 1 (HAP1) cells were plated in Iscove’s Modified Dulbecco’s Medium (IMDM; Thermo Fisher, Waltham, MA, USA; 21980065) and supplemented with 10% fetal bovine serum, FBS (Sigma, St. Louis, MO, USA; F7524), and 1% penicillin-streptomycin (Thermo Fisher, Waltham, MA, USA; 15140122) at 37 °C with 5% CO_2_, according to the manufacturer’s instructions. *MDC1^∆^* HAP1 cells are nearly haploid human cells that were custom-generated by request and provided by Horizon Discovery (Waterbeach, Cambridge, UK; HZGHC005077c003). The HAP1 cells are human, nearly haploid cell lines derived from the chronic myelogenous leukemia (CML) cell line (KMB-7). The HAP1 model has been recently used to develop knockout human cells (e.g., References [13,33,51,54]).

Both vAbl and HAP1 cells were counted every 24 h using a Countess™ Automated Cell Counter (Invitrogen, Carlsbad, CA, USA) with Trypan blue staining (Invitrogen, Carlsbad, CA, USA) and bright-field detection. Statistical analyses were performed using GraphPad Prism 8 (La Jolla, CA, USA), one-way analysis of variance (ANOVA), and *t*-test.

## 3. Results

### 3.1. Robust V(D)J Recombination in Progenitor-B Cells Lacking Mediator of DNA Damage Checkpoint Protein 1 (MDC1)

Mice lacking MDC1 possess nearly wild-type levels of B and T lymphocytes [40]. Combined inactivation of *MDC1* and *Artemis* suggests that MDC1 protects or stabilizes RAG-induced DSBs before ligation. In particular, the vAbl cells lacking MDC1 and Artemis possess ATM-dependent degradation of free DNA ends during the attempted V(D)J recombination [55]. To further determine the impact of MDC1 on the V(D)J recombination, we inter-crossed *MDC1^+/−^Eμ-Bcl2^+^* mice and isolated the cells from the bone marrow of three-week-old *MDC1^−/−^Eμ-Bcl2^+^* animals. We then established Abelson murine leukemia virus kinase-transformed pro-B cells (vAbl) and chromosomally-integrated either *pMX-INV* or *pMX-DEL* V(D)J recombination cassettes, as described previously [18,34,42,43,49]. Similar to wild type (WT) controls, two independently generated MDC1-deficient vAbl cell lines possessed robust coding-end (CE) and signal end (SE) joining (Supplementary Figure S1). We concluded that MDC1 is dispensable for the V(D)J recombination in WT vAbl progenitor B cells.

### 3.2. Synthetic Lethality Between Mediator of DNA Damage Checkpoint Protein 1 (MDC1) and XRCC4-Like Factor (XLF) in Mouse

To further investigate the role of MDC1 during the V(D)J recombination, we first attempted to generate the *MDC1^−/−^XLF^−/−^* double knockout mice. Individual inactivation of *MDC1* or *XLF* results in live-born mice that possess modest levels of DNA repair defects [24,25,34,40,42,43,44]. First, we obtained *MDC1^+/−^XLF^−/−^* mice, starting with available heterozygous *MDC1^+/−^* [40] and *XLF^+/−^* [24] animals. By inter-crossing *MDC1^+/−^XLF^−/−^* mice, we obtained and genotyped 104 pups, including 34 *MDC1^+/+^XLF^−/−^* and 70 *MDC1^+/−^XLF^−/−^* (Table 1). Strikingly, we detected no *MDC1^−/−^XLF^−/−^* double knockout pups, and the final genotype distribution was 34:70:0 (1:2:0) (Table 1). We concluded that combined inactivation of *MDC1* and *XLF* results in embryonic lethality.

### 3.3. Generation of XLF^−/−^ Knockout and MDC1^−/−^XLF^−/−^ Double Knockout vAbl Cell Lines

**Figure 1:**
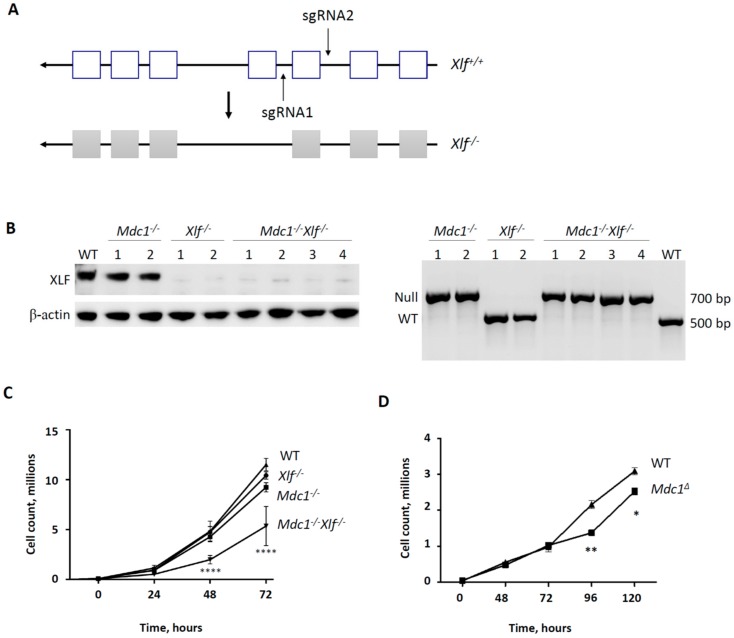
Generation of *MDC1^−/−^XLF^−/−^* vAbl cells. (**A**) Clustered regularly interspaced short palindromic repeats (CRISPR)/CRISPR-associated protein 9 (Cas9)-mediated inactivation of the *XLF* gene targeting *exon 3* in Abelson murine leukemia virus-transformed (vAbl) progenitor-B cell lines. (**B**) Western blot detecting XRCC4-like factor (XLF) protein in wild type (WT) and Mediator of DNA Damage Checkpoint Protein 1-deficient (*MDC1^−/−^)* vAbl cells. No signal corresponding to XLF was detected in *XLF^−/−^* and *MDC1^−/−^XLF^−/−^* vAbl cells. Antibody against beta-actin was used to detect beta-actin, a loading control (left). Polymerase chain reaction (PCR) followed by agarose gel electrophoresis detecting *MDC1* null and WT alleles (right). The 500 base pairs (bp) band corresponds to the WT allele, and the 700 bp band corresponds to the *MDC1* null allele (right). (**C**) The proliferation of vAbl cells lacking either XLF or MDC1, both MDC1/XLF, and WT controls. WT, *XLF^−/−^*, and *MDC1^−/−^* cells proliferate with a similar rate (n.s., *p* > 0.05). *MDC1^−/−^XLF^−/−^* cells proliferate slower than WT, *XLF^−/−^* and *MDC1^−/−^* vAbl cells (****, *p* < 0.0001). Data represent the mean ± standard deviation (SD) of three independent experiments using 1 WT control, 2 *MDC1^−/−^*, 2 *XLF^−/−^,* and 2 *MDC1^−/−^XLF^−/−^* clones. (**D**) The proliferation of haploid 1 (HAP1) cells lacking MDC1, and wild type (WT) controls. *MDC1^∆^* HAP1 cells possess reduced proliferation rates when compared to WT at 96 and 120 hours (h) of the experiment (*, *p* < 0.05; **, *p* < 0.01). Data represent the mean ± standard deviation (SD) of three independent experiments using WT parental control and *MDC1^∆^* clones.

To obtain double knockout *MDC1^−/−^XLF^−/−^* and control *XLF^−/−^* vAbl cells, we inactivated the *XLF* gene in *MDC1^−/−^* and WT vAbl cells using the CRISPR/Cas9 gene-editing approach (see the Materials and Methods Section). Briefly, we targeted *exon 3* of the *XLF* gene (Figure 1A) and verified gene inactivation by western blot (Figure 1B) and DNA sequencing (available upon request). The proliferation of WT, *XLF^−/−^*, and *MDC1^−/−^* vAbl cells were of similar rates during the 72 h, *p* > 0.05 (Figure 1C). On the contrary, *MDC1^−/−^XLF^−/−^* double knockout vAbl cell lines possessed reduced proliferation rates (****, *p* < 0.0001) at 48 and 72 h of the experiment (Figure 1C). Inactivation of the *MDC1* gene in human HAP1 cells resulted in proliferation rates similar to WT cells at 24–72 h, and reduced proliferation rates at 96 and 120 h (Figure 1D).

### 3.4. Reduced V(D)J Recombination Efficiency in vAbl Pro-B Cells Lacking both MDC1 and XLF

**Figure 2:**
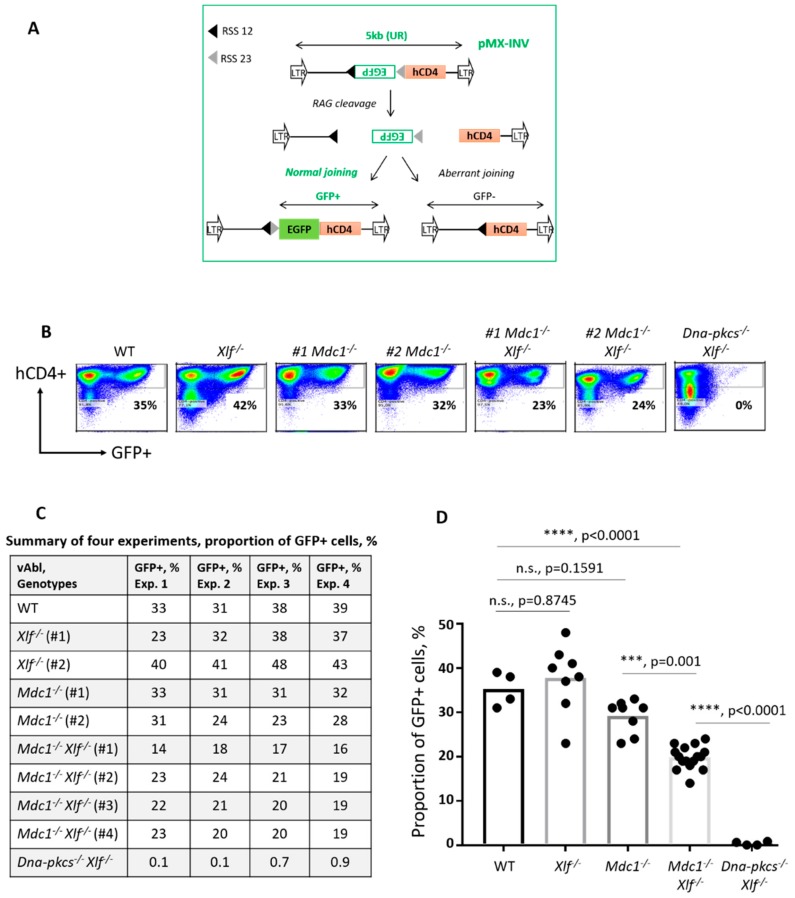
Mediator of DNA Damage Checkpoint Protein 1 (MDC1) stimulates the *variable (V)*, *diversity (D)* and *joining (J)* gene segments recombination (V(D)J recombination) in the cells lacking XRCC4-like factor (XLF). (**A**) Schematic representation of the green fluorescent protein (GFP) expression-based V(D)J recombination reporter. Upon treatment with STI571, the recombination activating gene (RAG) induces DNA double-strand breaks (DSBs) at dedicated sites flanking the *GFP* gene in reverse orientation. After inversion and DSB repair, the *GFP* gene is placed in the sense orientation, and the GFP protein is expressed and detected by flow cytometry. (**B**) Examples of flow cytometry-based quantification of GFP-positive vAbl cells (WT, *XLF^−/−^, MDC1^−/−^, MDC1^−/−^XLF^−/−^*, and *DNA-PKcs^−/−^XLF^−/−^*) following exposure to STI571 for 96 hours (h). The human cluster of differentiation 4 (hCD4) was used as a surface marker of the chromosomally integrated V(D)J recombination cassette. At day 0, vAbl cells were sorted based on the hCD4 expression, and hCD4-positive cells were used for the experiments. (**C**) Proportions of the GFP-positive vAbl cells of indicated genotypes in the V(D)J recombination experiments using chromosomally integrated cassettes. Data represent the mean ± standard deviation (SD) of four independent experiments using one WT, two *XLF^−/−^*, two *MDC1^−/−^,* and four *MDC1^−/−^XLF^−/−^* lines, used in all the experiments. *DNA-PKcs^−/−^XLF^−/−^* vAbl cells were used as a non-homologous end joining (NHEJ)-deficient negative control, to establish background levels of GFP expression. (**D**) Statistical analyses of V(D)J recombination efficiency in vAbl cells. WT versus *XLF^−/−^* (n.s., *p* = 0.8745); WT versus *MDC1^−/−^* (n.s., *p* = 0.1591), WT versus *MDC1^−/−^XLF^−/−^* (****, *p* < 0.0001); WT versus *DNA-PKcs^−/−^XLF^−/−^* (****, *p* < 0.0001); *XLF^−/−^* versus *MDC1^−/−^XLF^−/−^* (****, *p* < 0.0001); *XLF^−/−^* versus *DNA-PKcs^−/−^XLF^−/−^* (****, *p* < 0.0001); *MDC1^−/−^* versus *MDC1^−/−^XLF^−/−^* (***, *p* = 0.0001); *MDC1^−/−^* versus *DNA-PKcs^−/−^XLF^−/−^* (****, *p* < 0.0001); *MDC1^−/−^XLF^−/−^* versus *DNA-PKcs^−/−^XLF^−/−^* (****, *p* < 0.0001).

To determine the impact of MDC1 on V(D)J recombination, we chromosomally-integrated the cassette-carrying *GFP* gene in reverse orientation and flanked by DNA sequences recognized by RAG (*pMX-INV*) [49,50] (Figure 2A). To induce the RAG expression, we exposed the cells to the vAbl kinase inhibitor STI571 (Gleevec). Upon a successful V(D)J recombination event, the cells expressing GFP were detectable by flow cytometry [42,49,50]. The cells lacking MDC1 possessed relatively high levels of V(D)J recombination reflected by GFP expression (29%), which was in the range of WT and *XLF^−/−^* cell lines (34% and 37%, respectively) (Figure 2B–D). Strikingly, combined inactivation of *MDC1* and *XLF* resulted in a significantly reduced proportion of GFP-expressing vAbl cells when compared to WT and single knockout controls (average levels of 20%; ****, *p* < 0.0001). Double knockout *DNA-PKcs^−/−^XLF^−/−^* vAbl cells were used as a negative control to establish background levels of the experiments (0% of GFP-positive cells) [34]. We concluded that MDC1 is stimulating the V(D)J recombination in XLF-deficient cells, due to functional complementarity between MDC1 and XLF in this process.

## 4. Discussion

Inactivation of *RAG* and most of the known NHEJ factor genes in mice leads to immunodeficiency [12,56]. Recently, we and others found that single inactivation of *XLF*, *PAXX*, or *Mri* genes results in mice with the nearly normal immune system, due to the overlapping functions between XLF and PAXX [26,27,28,29,33], as well as XLF and Mri [12,13] (Table 2). The ATM-dependent DDR pathway was initially thought to be dispensable for the V(D)J recombination, although more recent studies using combined genetic inactivation of *XLF* and *ATM* [42], as well as *DNA-PKcs* and *ATM* [21,57], revealed that ATM is indeed involved in the early stages of B and T lymphocyte development and its function is partially compensated by XLF and DNA-PKcs. Later, we and others found that ATM substrates, H2AX and 53BP1, are also required for B and T lymphocyte development due to their functions in V(D)J recombination [42,43,44] (Table 2). Here, we show that another ATM substrate, MDC1, is involved in the V(D)J recombination and its function is compensated in WT cells by XLF. Combined inactivation of *ATM* and *XLF*, or *53BP1* and *XLF*, resulted in immunodeficient mice of smaller sizes than single knockouts or wild-type controls, with abrogated NHEJ, resembling *Ku70^−/−^* or *Ku80^−/−^* knockouts [1,42,43,44]. Differently, combined inactivation of *DNA-PKcs* and *XLF* [34,35], *H2AX* and *XLF* [42], or *MDC1* and *XLF* ([33]; and this study) resulted in embryonic lethality in mice, challenging genetic interaction studies in vivo (Table 2). One option to overcome this obstacle is to develop conditional knockouts allowing inactivation of *DNA-PKcs*, *XLF*, or *MDC1* in developing B and T lymphocytes in adult mice. An alternative option is to develop more complex mouse models using. for example, *p53^−/−^* or *p53^+/−^* backgrounds, allowing for the rescue of embryonic lethality (e.g., References [33,35]).

Knocking out genes of interest in cell lines may complement and sometimes substitute in vivo experiments using transgenic mice. In particular, vAbl cell lines can be modified using the CRISPR/Cas9 gene-editing approach and serve as a model system to elucidate the specific roles of a particular gene (e.g., References [30,31,32,50]). Moreover, human, nearly haploid HAP1 cells derived from the KMB-7 cell lines have been recently used to develop genetically-modified cells (e.g., References [13,33,51,54]).

It becomes more accepted that the DDR pathway contributes to the V(D)J recombination in developing B and T lymphocytes [1,34,42,43,44]. However, the mechanistic aspects underlying the specific roles of the DDR factors in this process remain unclear. One can speculate that DDR factors share the functions with XLF, e.g., by stabilizing the DNA repair complex or supporting timely recruitment and dissociation of the NHEJ factors. The DDR pathway may also contribute to distinct but complementary XLF aspects of the DNA repair, e.g., by recruiting the downstream enzymes, supporting the DNA damage-induced post-translational modifications of DNA repair factors and histones, or protecting the free DNA ends from the nuclease-dependent processing before the DNA ligation step [1,34,42,43,44,55]. In particular, the role of MDC1 during the V(D)J recombination might be to stabilize the DNA repair complex, to protect the free DNA ends, to ensure efficient recruitment of downstream DDR factors, such as 53BP1, PTIP, RIF1, Shieldin, etc. [1,42,43,44,47,55,58], or to exit from the G1 phase of the cell cycle following the RAG-induced DSB [59]. Further research is required to identify specific roles of MDC1 and XLF in DNA repair.

The proliferation rate of vAbl cells lacking both XLF and MDC1 was reduced when compared to single-deficient and WT controls (Figure 1) at 72 h. Moreover, proliferation rates of MDC1-deficient cells were also reduced when compared to WT, although not significant. Furthermore, the lack of MDC1 alone resulted in significantly reduced proliferation rates of human HAP1 cells at 96 and 120 h (Figure 1). These observations may suggest that, first, the lack of MDC1 is compensated by the presence of XLF in murine cells, and second, that the MDC1 is required for efficient DNA repair and proliferation of human cells, likely by supporting the cell cycle progression and DNA damage tolerance [47,59].

## 5. Conclusions

Multiple DDR factors are involved in the V(D)J recombination. Due to the functional redundancy between the DDR and NHEJ pathways, complex genetic in vivo and in vitro models will be appropriate to uncover specific functions of DDR factors in B and T lymphocyte development and further elucidate mechanisms underlying their roles.

## Figures and Tables

**Table 1:** Synthetic lethality between Mediator of DNA Damage Checkpoint Protein 1 (*MDC1*) and XRCC4-like factor (*XLF*).

Genotypes	Live Born	Expected (1:2:1)	Expected * (1:2:0)
*MDC1^+/+^XLF^−/−^*	34	26	35
*MDC1^+/−^XLF^−/−^*	70	52	69
*MDC1^−/−^XLF^−/−^*	0	26	0
**Total**	**104**	**104**	**104**

* Corrected expected distribution, which does not include the probability of *MDC1^−/−^XLF^−/−^* mice.

**Table 2:** Impact of NHEJ-deficiency on V(D)J recombination in mice.

Genotypes	V(D)J Recombination	Mice
**Single Knockouts**		
*DNA-PKcs^−/−^* [23]	No	Alive
*PAXX^−/−^* [9,10,11,27]	Normal	Alive
*Mri^−/−^* [12,13]	Normal	Alive
*XLF^−/−^* [24,25]	Normal	Alive
*ATM^−/−^* [37]	Normal	Alive
*H2AX^−/−^* [38,39]	Reduced	Alive
*MDC1^−/−^* [40]	Normal	Alive
*53BP1^−/−^* [41]	Normal	Alive
*RAG2^Δ/Δ^* [60]	Reduced	Alive
**Double Knockouts**		
*XLF^−/−^ DNA-PKcs^−/−^* [34,35]	No	Embryonic lethality
*XLF^−/−^ PAXX^−/−^* [26,28,29,33]	No	Embryonic lethality
*XLF^−/−^ Mri^−/−^* [12]	No	Embryonic lethality
*XLF^−/−^ ATM^−/−^* [42]	Very low	Alive, small
*XLF^−/−^ H2AX^−/−^* [42]	Reduced	Embryonic lethality
*XLF^−/−^ MDC1^−/−^* [*]	Reduced	Embryonic lethality
*XLF^−/−^53BP1^−/−^* [43,44]	Very low	Alive, small
*XLF^−/−^ RAG2^Δ/Δ^* [36]	Very low	Alive

References are cited. This study [*].

## Supplementary Materials

The following are available online at https://www.mdpi.com/2218-273X/10/1/60/s1, Figure S1: Robust V(D)J recombination in vAbl cells lacking MDC1.
