# Supplementary material for: Mediator of DNA Damage Checkpoint Protein 1 Facilitates V(D)J Recombination in Cells Lacking DNA Repair Factor XLF
Source: Biomolecules. 2019 Dec 30;10(1):60. doi: 10.3390/biom10010060 (PMC7023129; doi:10.3390/biom10010060)
Supplement: Supplementary file 1 [file biomolecules-10-00060-s001.pdf]

Mediator of DNA damage checkpoint protein 1 facilitates V(D)J recombination in cells lacking DNA repair factor XLF

Carole Beck [1,2], Sergio Castañeda-Zegarra [1,2], Camilla Huse [1,2], Mengtan Xing [1,2], Valentyn Oksenych [1,2,3,*]

[1] Department of Clinical and Molecular Medicine (IKOM), Norwegian University of Science and technology, 7491 Trondheim, Norway; carole.beck@ntnu.no (C.B.); camilla.huse@rr-research.no (C.H.); sergio.m.c.zegarra@ntnu.no (S.CZ.); valentyn.oksenych@ntnu.no (V.O.)

[2] St. Olavs Hospital, Trondheim University Hospital, Clinic of Medicine, Postboks 3250 Sluppen, 7006 Trondheim, Norway.

[3] Department of Biosciences and Nutrition (BioNuT), Karolinska Institutet, 14183 Huddinge, Sweden.

* Correspondence: valentyn.oksenych@ntnu.no;

This material includes: Robust V(D)J recombination in vAbl cells lacking MDC1 (Figure S1).

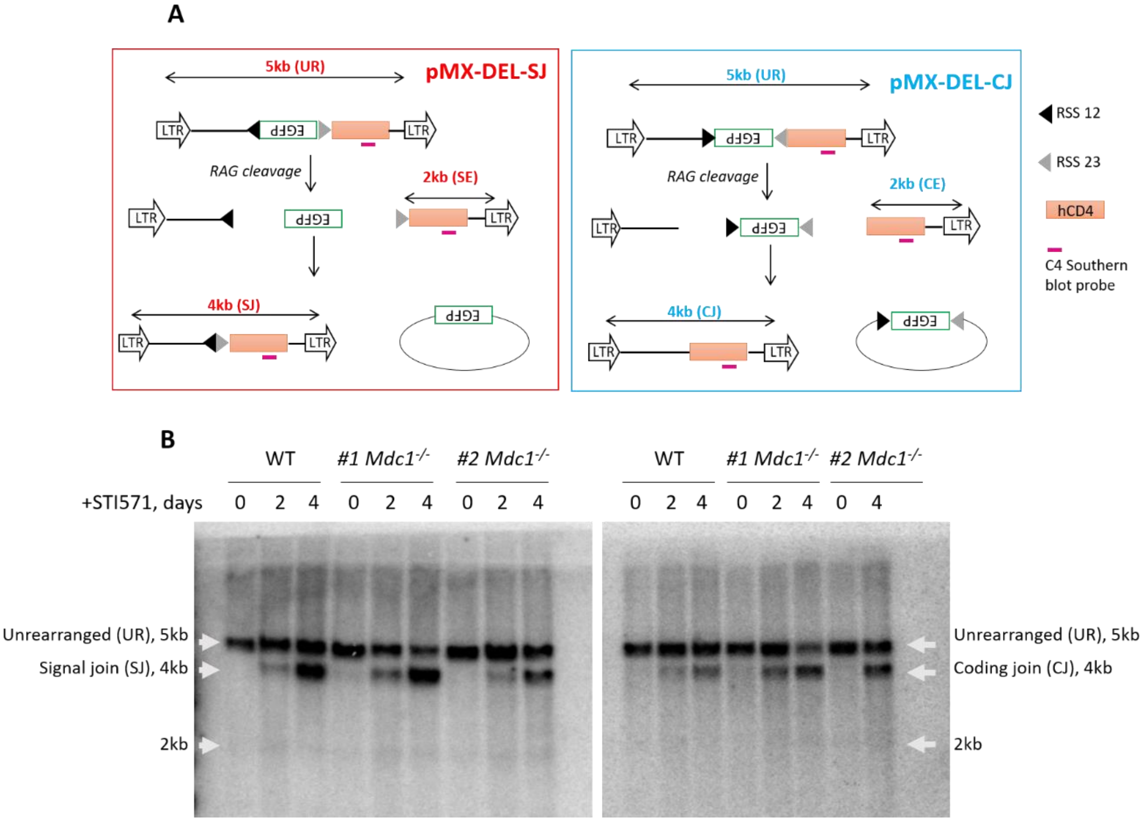

Figure S1. Robust V(D)J recombination in vAbl cells lacking MDC1. (A) Schematic representation of pMX-Del-SJ deletional V(D)J recombination cassette with blunt SJ DNA ends (left) and pMX-Del-CJ cassette with hairpin-sealed CJ DNA ends (right). (B) Southern blot representing original V(D)J recombination cassette (5kb), and product of deletional V(D)J rcombination (4kb). Very weak to no signal is detected at 2kb (free DNA ends) suggesting either rapid DNA repair or degradation of unjoined DNA ends.
